# A Self-Powered Transparent Photodetector Based on Detached Vertical (In,Ga)N Nanowires with 360° Omnidirectional Detection for Underwater Wireless Optical Communication

**DOI:** 10.3390/nano11112959

**Published:** 2021-11-04

**Authors:** Jianya Zhang, Min Jiang, Lifeng Bian, Dongmin Wu, Hua Qin, Wenxian Yang, Yukun Zhao, Yuanyuan Wu, Min Zhou, Shulong Lu

**Affiliations:** 1 School of Nano-Tech and Nano-Bionics, University of Science and Technology of China, Hefei 230026, China; jyzhang2019@sinano.ac.cn (J.Z.); dmwu2008@sinano.ac.cn (D.W.); mzhou2019@sinano.ac.cn (M.Z.); 2 Key Lab of Nanodevices and Applications, Suzhou Institute of Nano-Tech and Nano-Bionics (SINANO), Chinese Academy of Sciences (CAS), Suzhou 215123, China; mjiang2020@sinano.ac.cn (M.J.); lfbian2006@sinano.ac.cn (L.B.); hqin2007@sinano.ac.cn (H.Q.); wxyang2014@sinano.ac.cn (W.Y.); yywu2011@sinano.ac.cn (Y.W.); 3 School of Microelectronics, University of Science and Technology of China, Hefei 230026, China

**Keywords:** transparent photodetector, 360° omnidirectional detection, detached vertical (In,Ga)N nanowire, underwater communication, no power supply

## Abstract

Underwater wireless optical communication (UWOC) is a wireless communication technology using visible light to transmit data in an underwater environment, which has wide applications. Based on lift-off (In,Ga)N nanowires, this work has proposed and successfully demonstrated a self-powered photoelectrochemical (PEC) photodetector (PD) with excellent transmissivity. The transparent functionality of the PD is critical for 360° omnidirectional underwater detection, which was realized by detaching the (In,Ga)N nanowires from the opaque epitaxial substrates to the indium tin oxide (ITO)/glass. It was also found that the insulating SiO_2_ layer can enhance the photocurrent by about 12 times. The core–shell structure of the nanowires is beneficial for generating carriers and contributing to the photocurrent. Furthermore, a communication system with ASCII code is set to demonstrate the PD detection in underwater communication. This work paves an effective way to develop 360° omnidirectional PDs for the wide applications in UWOC system and underwater photodetection.

## 1. Introduction

On account of potential advantages of low latency and high safety, underwater wireless optical communication (UWOC) is considered as an important alternative candidate in addition to acoustic communications and radio frequency (RF) communications [1]. Meanwhile, underwater communication is significant for future applications in oceanography exploration and detection activities, such as marine resource exploration and environmental monitoring [1,2,3]. It is well known that seawater has a low attenuation of light waves in the wavelength range from 450 to 550 nm, i.e., the blue–green light transmission window [2,4,5]. Furthermore, the band gap of (In,Ga)N is direct and adjustable, which can cover the wavelength range from 365 to 1700 nm [6]. That means (In,Ga)N with appropriate In composition can be used for underwater communication. In addition to the extraordinary characteristics of being nontoxic, long lifetime and high stability against radiation and electrochemical (EC) etching [7,8], (In,Ga)N is an ideal material for making photodetectors (PDs) for UWOC applications.

Current available (In,Ga)N PDs are mostly fabricated based on thin film and bulk materials [6]. Compared with thin films or bulk materials, nanostructures such as nanowires (NWs) have the ability to significantly increase the optical absorption and photogenerated carrier density due to the larger surface-to-volume ratio [6,9]. Moreover, NWs have higher transparency compared to the films with the same thickness [7,10]. Up to now, (In,Ga)N material was normally grown on opaque substrates, such as sapphire, Si and SiC, which cannot meet the requirements for transparent devices [7,10], let alone 360° omnidirectional detection. Therefore, (In,Ga)N NWs are promising but highly challenging to be applied in transparent PDs.

Self-powered PD is an essential part of a photoelectric sensing and communication system, which can continuously work without additional power sources [11,12,13]. Due to the outstanding advantages of eco-friendly, low cost and simple fabrication processes, self-powered photoelectrochemical (PEC) PDs have attracted extensive attention [12,14,15]. In another aspect, transparent optoelectronic devices provide many novel functions and have the potential for many applications, such as wearable intelligent electronics, imaging device, etc. [7,10,13]. With the increase in detection demand, omnidirectional detection has become a hot research topic. Many novel PD applications require detecting light from 360° angles, such as surveillance cameras, optical tracing systems and optical field measurements [13,16,17]. However, for opaque plane PDs, it can only detect incident light in 180° hemispherical space, not in 360° omnidirectional space [16]. In order to achieve 360° detection, the common solution is to add other optical elements to the optical path, which not only increases the economic cost and optical intensity loss, but also makes the whole system more complicated [16]. Therefore, the development of self-powered PDs with good transparency is very important to meet the ever-changing needs of the next generation of photoelectric devices.

In this work, we detached (In,Ga)N NWs from silicon (Si) substrates to make transparent self-powered PEC PDs successfully. We also demonstrated the application of self-powered PD with high transparency in an underwater communication system. Most notably, the proposed self-powered PD has been demonstrated to have the ability of 360° omnidirectional detection. We also found that such PD has the advantage of determining the direction of photons detected.

## 2. Experiments and Methods

### 2.1. Preparation of (In,Ga)N NWs

**Figure 1:**
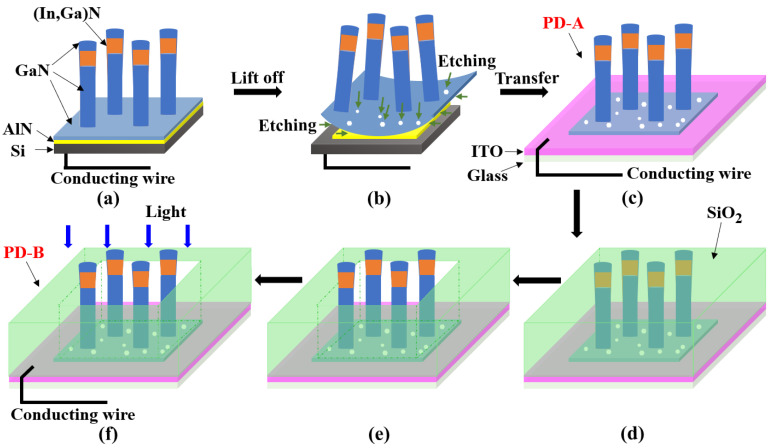
Schematic illustration of fabricating the PEC PD. (**a**) MBE growth of (In,Ga)N NWs on Si substrate; (**b**) use the EC etching to detach (In,Ga)N NW film from the epitaxial substrate; (**c**) transfer the NW film to an ITO/glass substrate; (**d**) deposit the SiO_2_ dielectric layer; (**e**) selectively etch the SiO_2_ layer to expose the NWs to air; (**f**) connect the PD with conducting wire and measure it under illumination.

Molecular beam epitaxy (MBE, Vecco G20, St. Paul, MN, USA) was utilized to prepare (In,Ga)N NWs on n-type Si(111) substrates (Figure 1a). Before growth, the Si substrates should be heated up to about 900 °C for 15 min in the growth chamber to eliminate native oxides by observing the 7 × 7 reconstruction. The NWs were grown in the growth chamber with the Ga, In effusion cells and an N plasma cell. Initially, an AlN buffer layer was grown with Al flux of ~3 nm/min for 1 min, which can be used as a sacrificial layer in the electrochemical etching process. The substrate temperature is set to be 830 °C. After that, GaN NWs were grown with a Ga flux of ~2 × 10^−8^ Torr for 120 min. After the growth of GaN section, the (In,Ga)N section was grown for a total time of 50 min. Then, the GaN segment was grown for 30 min with Ga flux of 2 × 10^−8^ Torr. This layer is used to cap and protect (In,Ga)N section.

### 2.2. Lift-Off Procedure of (In,Ga)N NW Films

First, the as-grown 2-inch NW sample was divided into small pieces. To make ohmic contact, In/Al/Au alloys were melted on the back side of Si substrate with a welding torch and leaded wire for circuit connection. To avoid the leakage current and EC corrosion, these electric contacts were coated with epoxy resin (Figure 1a). The EC etching process was carried out in an H-type cell. During the EC etching, the NW sample and Cu plate were used as the working and counter electrodes, respectively. Both electrodes were immersed in 1 mol/L sodium hydroxide (NaOH). After a certain time under an applied bias, the (In,Ga)N NWs were expected to be lifted off from the original Si and transferred to an ITO/glass substrate (Figure 1b,c).

### 2.3. Fabrication of Self-Powered (In,Ga)N PEC PD

After the lift-off process, the transferred samples were soaked in deionized water for about 20 min to remove the alkali solution. Later, a SiO_2_ layer was deposited on top of the NWs and ITO surface by the chemical vapor deposition (CVD, Figure 1d). Then the SiO_2_ film was selectively removed by photolithography and reactive ion etching (RIE) to expose the NWs to air (Figure 1e). The In/Al/Au alloy was melted on the edge of the ITO layer with a welding torch for electrical contact (Figure 1f).

### 2.4. Characterization and Measurement Methods

To characterize the NW morphology and element distribution, scanning electron microscopy (SEM) and scanning transmission electron microscopy (STEM) with high-resolution energy dispersive X-ray (EDX) mapping were used. A focused ion beam (FIB) was utilized to prepare the STEM samples. The optical properties were studied by photoluminescence (SP2500i, Princeton Instruments, Trenton, NJ, USA) and spectrophotometer (Lambda 750, Perkinelmer Instruments, Waltham, MA, USA). An electrochemical workstation (DH 7000, Jiangsu Donghua Analytical Instrument Co. Ltd., Taizhou, China) was used to evaluate the electrical properties of (In,Ga)N PD in an H-type cell, which was made of quartz with a high transparency in the visible range.

## 3. Results and Discussion

In order to systematically study the (In,Ga)N PEC PD, two kinds of samples were designed and prepared (Figure 1a–f and Table 1). Details of the material growth, device fabrication and characterization methods were described in the experiments and methods section previously. As shown in Figure 1a, the as-grown (In,Ga)N nanowires were prepared by molecular beam epitaxy. Then electrochemical etching (EC) was used to detach the nanowires from the original Si substrate (Figure 1b). We transferred the detached nanowires to the ITO/glass substrate to make sample PD-A (Figure 1c). In order to make a better comparison, we created the sample PD-B with SiO_2_ deposition and photolithography (Figure 1d–f). The PD-A meant the PEC PD did not have SiO_2_ to cover the ITO surface, while PD-B did. The detached nanowires and the transparent ITO/glass substrate together created our transparent device.

**Figure 2:**
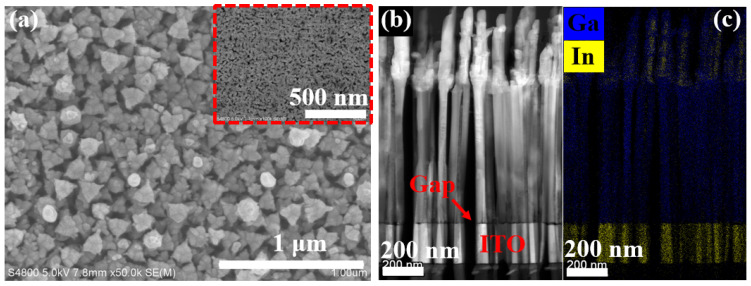
SEM/STEM characterizations of detached (In,Ga)N NWs. (**a**) Plan-view SEM image of the top of detached (In,Ga)N NWs. The inset is the plan-view SEM image of the bottom surface of detached GaN parasitic layer. (**b**) Side-view STEM image and (**c**) high-resolution EDX mapping of detached (In,Ga)N NWs.

As illustrated in Figure 2a, the netlike GaN parasitic layers at the bottom NWs were used to connect all NWs. Figure 2a,b show that the well vertically aligned (In,Ga)N NWs exhibit a dense arrangement on ITO substrate. The NW heights were around 1 μm. The diameters of bottom GaN NWs were about 40 nm, while those of (In,Ga)N section were around 70 nm. Furthermore, the NW bottom (GaN parasitic layer) was smooth with a small, flat gap between the (In,Ga)N NWs and the ITO layer. This small and smooth gap was conducive to effectively enhancing the carrier transport capacity, thus improving PD response performance. Figure 2c shows that the NW structure agrees well with the epitaxial design.

**Figure 3:**
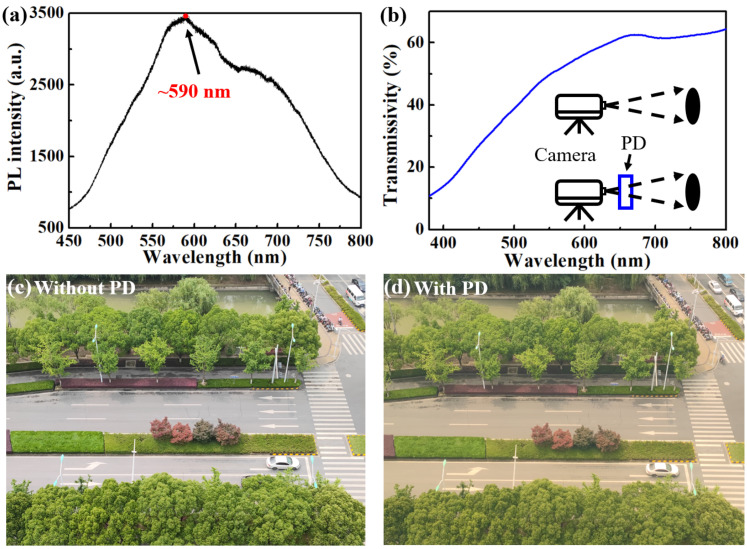
(**a**) PL spectra of (In,Ga)N NWs measured at room temperature; (**b**) transmission spectra of PD-B. The inset is the schematic illustrations of taking images through PD. Take the optical images (**c**) without PD and (**d**) with PD-B.

To characterize the composition of the In component, a photoluminescence (PL) curve is illustrated in Figure 3a, which had a peak of ~590 nm. The In composition was calculated by Vegard’s law [6]. The band gap of InN was 0.7 eV and that of GaN was 3.4 eV [6]. The bowing parameter of (In,Ga)N is often selected as 1.43 eV. After calculation, the In composition corresponding to the PL peak wavelength of 590 nm was about 36%. As clearly illustrated in Figure 3b, the maximum transmissivity of PD was over 60%, which is high compared with the most-reported GaN-based devices (Table 2). Due to the high transparency, the PD could be used for the omnidirectional detection [7,18,19,20,21]. To intuitively demonstrate the transparency, we used a camera as Figure 3b to take pictures through the PDs. As the pictures show in Figure 3c,d, no obvious changes were obtained between the two images with and without the PDs, which demonstrates the excellent transparent performance of our PEC PDs.

To evaluate the photoelectric conversion characteristics of the self-powered PEC PDs, we measured the photocurrent (*I*_ph_) at zero-bias voltage, and calculated the photocurrent densities (*J*_ph_) as following equation [14,24,25]:

*I*_ph_*= I*_light_ − *I*_dark_, (1)

**Figure 4:**
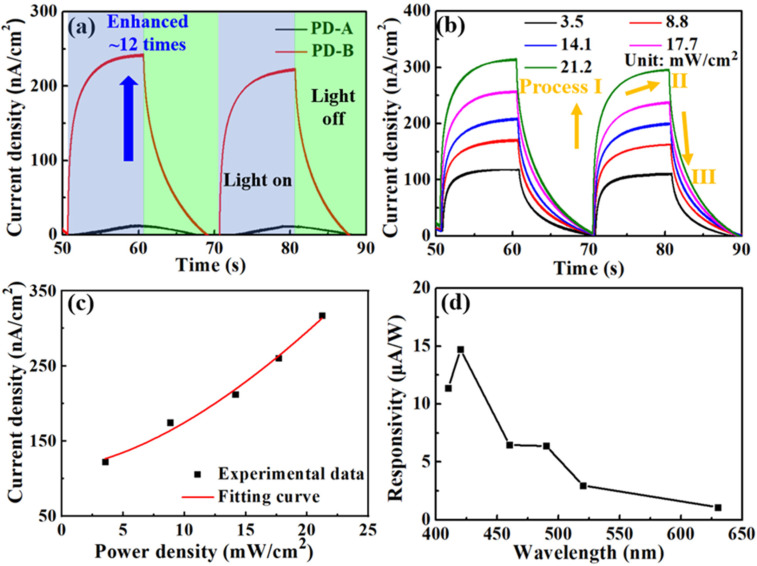
(**a**) Photo-switching behaviors of the self-powered PDs under 420 nm illuminations; (**b**) photocurrent densities of PD-B illuminations with different light powers. The unit of incident light power density is mW/cm^2^; (**c**) photocurrent density as a function of power density at 420 nm; (**d**) responsivity of PD-B at different wavelengths of illumination light.

*J*_ph_ = *I*_ph_/*S*, (2)

where *I*_light_ is the current with illumination and the *I*_dark_ is the current without illumination. *S* is the area of NW film (Figure 1c). As illustrated in Figure 4a, *J*_ph_ of the PD-B was much higher (~12 times) than that of the PD-A, indicating the importance of the SiO_2_ layer. From Figure 4b, the current under 420 nm illumination exhibited regular on–off behavior due to the photogenerated carriers. An overall upward shift can be obtained when increasing the light power density illuminating on the PD-B. Moreover, the quantitative dependence of the steady state photocurrent on the power density of incident light is plotted in Figure 4c, which can be fitted by the following equation [13,25]:

(3) $$J_{\mathrm{ph}}\propto P^{\beta }.$$

Here, *P* is the power density of incident light from the commercial light-emitting diode (LED) and *β* is a factor to determine the response. By fitting, *β* of PD-B is 1.6. The photocurrent density tends to saturate gradually with the increase in power density. Moreover, to clarify how the photocurrents affected by different polarization angles (*α*), we added a polarizer between the incident light source and PD (Figure S1a). It is clearly shown in Figure S1b that the measured photocurrents of PD-B at different *α* remained stable. Thus, the polarization angle is proposed to have a limited effect on the photocurrent. After continuously working around 3 h, the photocurrent of PD-B was essentially unchanged (Figure S2). Hence, PD-B exhibits an excellent stability. As the key evaluation parameter for photodetectors, the responsivity of our device is calculated as [13]:

*R* = *J*_ph_/*P*. (4)

As shown in Figure 4d, PD-B had an obvious response in the wavelength range smaller than 520 nm. The (In,Ga)N NWs with ~36% In composition can absorb the photons with the wavelengths smaller than 520 nm. Therefore, the photocurrent and responsivity are mainly attributed to the response of detached (In,Ga)N NWs within PD-B.

**Figure 5:**
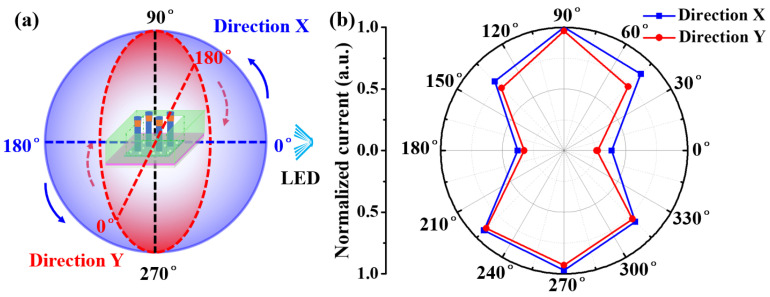
(**a**) Schematic illustration of the method for omnidirectional (360°) detection; (**b**) relative responsivity of the transparent PD-B with different rotation angles.

Due to the high transparency, the PD can be used in the all-round detection of 360 degrees. In order to better display the all-round detection capability of the PD, we measured the angular responsivity at different incident angles (Figure 5a) under the 420 nm illumination of LED. “Direction X” and “Direction Y” represent the two detection planes, which lie on two vertical intersecting planes with the center of the sphere. Figure 5b shows the responsivity along Direction X and Direction Y at intervals of 45 degrees. Under periodic on/off switching illumination light, the responsivity increased gradually from the minimum value (0°, incident light parallel to PD) to maximum value (90°, incident light perpendicular to PD), and then decreased to minimum value (180°, incident light parallel to PD again). The trend of responsivity variation with the angle of the illumination light in the back field (180°~360°) of PD is similar with that in the front field (0°~180°) of PD. As the ITO/glass and GaN parasitic layers are highly transparent in the visible range, our (In,Ga)N self-powered PEC PD can achieve 360° quasi-nondestructive detection underwater.

**Figure 6:**
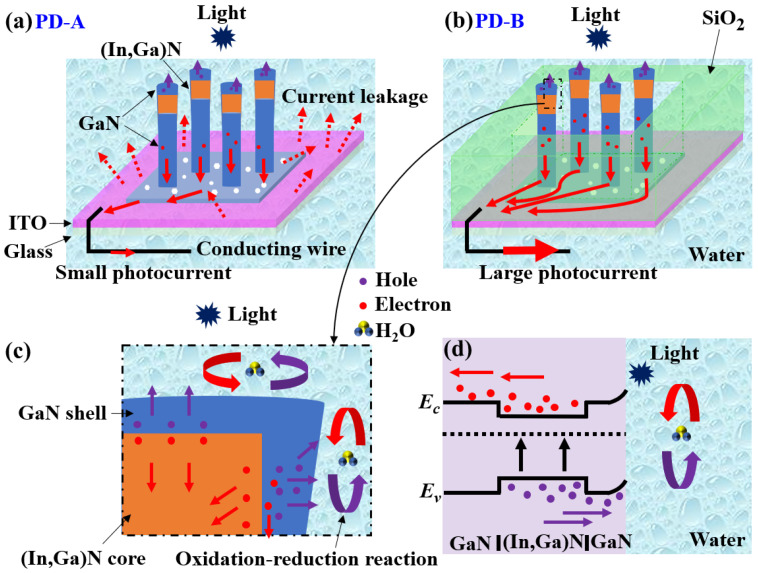
Schematic illustrations of (**a**) PD-A; (**b**) PD-B; (**c**) the enlarged (In,Ga)N core–shell structure, and (**d**) the corresponding energy band diagram under illuminations.

The working principles of the PEC PDs are analyzed by the schematic illustrations plotted in Figure 6. For PD-A (without SiO_2_ layer), as the ITO was conductive and connected to water directly (Figure 6a), the photocurrent easily leaked from the ITO surface. Thus, only a small amount of photogenerated carriers could be collected by the conducting wire (Figure 6a). In order to solve the leakage current problem, we used the method of depositing SiO_2_ as an insulating layer (PD-B). As a result, much more carriers can be collected by the conducting wire, leading to a much higher photocurrent (Figure 6b). Considering the enhancement of ~12 times (Figure 4a), the current leakage of PD-A should be a major part of the photocurrent. Furthermore, depositing SiO_2_ on the PD-B surface can also strengthen the Van der Waals’ force connection between the GaN parasitic layer and ITO surface, which is beneficial to improve the stability of the whole PD.

From Figure 6c, the top NW surfaces can absorb photons and the photogenerated electron–hole carriers can transport in the vertical direction. Except for the top surface, the (In,Ga)N NW sidewall can also absorb photons and generate carriers, which could transport along the horizontal direction. The core–shell structures could provide both vertical and horizontal directions for carrier transport. Therefore, compared with planar structures, NWs have the ability to increase the optical absorption and photogenerated carrier density due to their larger surface-to-volume ratio. In addition, photogenerated carriers are excited from the valence band (*E_V_*) to the conduction band (*E_C_*) under illuminations, producing electron–hole pairs. When the (In,Ga)N section is in contact with the electrolyte (water), excess carriers are transported from the NWs to the electrolyte, establishing EC equilibrium at the NW and electrolyte interface (Figure 6d). The band bending at the interface between (In,Ga)N NWs and electrolyte accelerates the charge flow between photogenerated carriers and electrolyte ions. Photocurrents may be generated by the following reactions [14,15,26,27]:

4H^+^ + 4e^−^ = 2H_2_, (5)

4h^+^ + 2H_2_O = O_2_ + 4H^+^. (6)

When the illumination light is on, the current shifts positively (Process I in Figure 4b). This positive current indicates that photogenerated holes transport to water while the electrons transport to bottom NWs. As time goes on, the current density gradually increases to a new steady state under continuous illumination (Process II). When the light is off, electrons transport to the top NWs (Process III), which is the opposite direction of process I. In the process of charge transfer, the entire circuit has the characteristics of light collection and carrier transport without external bias. The optimization of NW density, size and energy band can pave a way to further improve the carrier transport and response, which needs to be further studied. 

**Figure 7:**
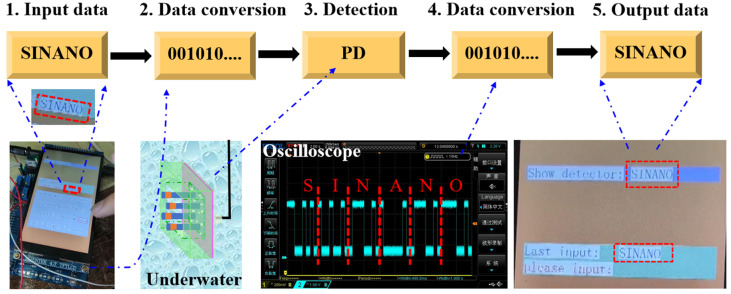
Proof of the underwater communication system based on the self-powered PEC PD. The system contains the display interface and the control panel with an electrical circuit for inputting and converting signals by ASCII codes.

In order to further demonstrate the PD performance of underwater detection, the self-powered PD-B is used as the receiver of the electrical signal and sets up the communication system as shown in Figure 7. The control panel with the electrical circuit utilizes ASCII code for converting signals of the letters “SINANO” in the communication system (Table S1). In order to ensure the feasibility and accuracy of digital communication, the input signal “SINANO” is converted into binary data through international ASCII code and output into optical signal by a program-controlled blue LED switch, which is clearly detected by PD and displayed in oscilloscope. Finally, the decoder decodes the oscilloscope waveform precisely to obtain the output signal of “SINANO” and displays it on the color display (Video S1). The whole process strongly demonstrates the feasibility of the (In,Ga)N PEC PD for underwater communication systems. In further study, a nanoslit metasurface could be utilized to increase the directivity of the transmitting beams and signal-to-interference ratio (SIR) for heterogeneous communication networks [28,29,30]. To sum up, the communication system based on the self-powered (In,Ga)N PEC PD shows great potential in underwater detection and visible light communication.

## 4. Conclusions

In this work, a self-powered PEC PD with omnidirectional underwater detection based on (In,Ga)N NWs was fabricated successfully. The opaque epitaxial substrate was detached using an electrochemical method to improve the transparency of the detector and achieve 360° omnidirectional underwater detection. By depositing the SiO_2_ insulating layer, the leakage current can be suppressed and the photocurrent density of the PD can be significantly increased by about 12 times. A communication system was established to verify the capability of underwater optical communication of the device. Therefore, this self-powered PEC PD has broad application prospects in underwater navigation and communication systems where low cost, low power consumption and 360° omnidirectional detection are required.

## Figures and Tables

**Table 1:** Overview of the NW samples used for this work.

Sample	Parameters	PEC PD	SiO_2_ Layer
PD-A	Detached NWs	Yes (Figure 1c)	No
PD-B	Detached NWs	Yes (Figure 1f)	Yes

**Table 2:** Comparison of the transmissivity between this work and other reported GaN-based devices.

Device	Wavelength (nm)	Transmissivity (%)	Ref.
Self-powered PD	650 800	62 64	This work
NW LED	600	63	[22]
GaN lens made by RIE	420	51	[23]

## Data Availability

The data presented in this study are available on request from the corresponding author.

## Supplementary Materials

The following are available online https://www.mdpi.com/article/10.3390/nano11112959/s1: Figure S1 shows the schematic diagram of a detection system and the relationship between the polarization angle of light source and the photocurrent of PD-B; Figure S2 demonstrates the photocurrent stability of PD-B; Table S1 shows the ASCII codes for related letters; Video S1 demonstrates the self-powered communication.
