# Supplementary material for: A Self-Powered Transparent Photodetector Based on Detached Vertical (In,Ga)N Nanowires with 360° Omnidirectional Detection for Underwater Wireless Optical Communication
Source: Nanomaterials (Basel). 2021 Nov 4;11(11):2959. doi: 10.3390/nano11112959 (PMC8621864; doi:10.3390/nano11112959)
Supplement: Supplementary file 1 [file nanomaterials-11-02959-s001.zip › Supplementary Materials-nanomaterials-1417214/Supplementary Materials-nanomaterials-1417214.pdf]

Article

A Self-Powered Transparent Photodetector Based on Detached Vertical (In,Ga)N Nanowires with 360° Omnidirectional Detection for Underwater Wireless Optical Communication

Jianya Zhang [1,2], Min Jiang [2,3], Lifeng Bian [2], Dongmin Wu [1,2], Hua Qin [2], Wenxian Yang [2], Yukun Zhao [2,*], Yuanyuan Wu [2], Min Zhou [1,2] and Shulong Lu [1,2,*]

1 School of Nano-Tech and Nano-Bionics, University of Science and Technology of China, Hefei 230026, China; jyzhang2019@sinano.ac.cn (J.Z.); dmwu2008@sinano.ac.cn (D.W.); mzhou2019@sinano.ac.cn (M.Z.)
2 Key Lab of Nanodevices and Applications, Suzhou Institute of Nano-Tech and Nano-Bionics (SINANO), Chinese Academy of Sciences (CAS), Suzhou 215123, China; mjiang2020@sinano.ac.cn (M.J.); lfbian2006@sinano.ac.cn (L.B.); hqin2007@sinano.ac.cn (H.Q.); wxyang2014@sinano.ac.cn (W.Y.); yywu2011@sinano.ac.cn (Y.W.)
3 School of Microelectronics, University of Science and Technology of China, Hefei 230026, China
* Correspondence: ykzhao2017@sinano.ac.cn (Y.Z.); sllu2008@sinano.ac.cn (S.L.)

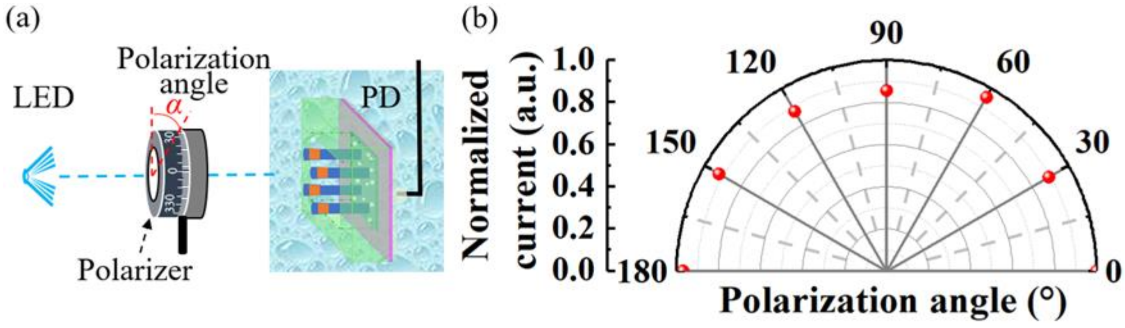

Figure S1. (**a**) Schematic diagram of a detection system with polarization angle (α). (**b**) The relationship between the polarization angle of light source and the photocurrent of PD-B.

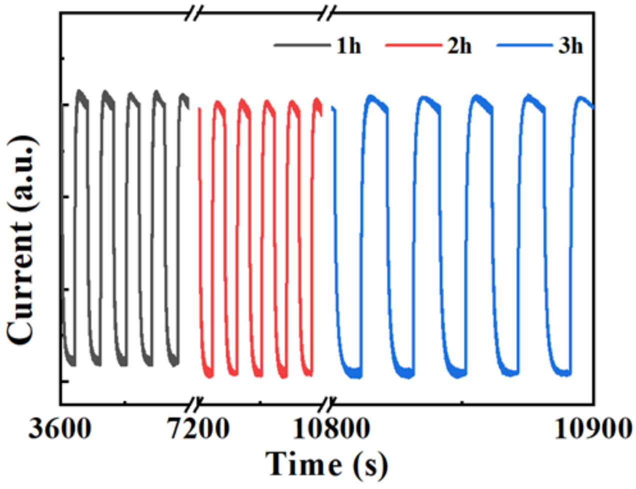

Figure S2. Photocurrent stability of PD-B under 420 nm illumination.

Table S1. ASCII codes for related letters.

Letter	ASCII code (low-order to high-order)
“S”	1100 1010
“I”	1001 0010
“N”	0111 0010
“A”	1000 0010
“N”	0111 0010
“O”	1111 0010

Video S1. Video of demonstrating self-powered communication.
